# A Homozygous Deep Intronic Mutation Alters the Splicing of Nebulin Gene in a Patient With Nemaline Myopathy

**DOI:** 10.3389/fneur.2021.660113

**Published:** 2021-06-15

**Authors:** Nathalie Laflamme, Baiba Lace, Samarth Thonta Setty, Nadie Rioux, Yvan Labrie, Arnaud Droit, Nicolas Chrestian, Serge Rivest

**Affiliations:** ^1^Centre de recherche CHU de Québec- Laval University, Quebec City, QC, Canada; ^2^Department of Medical Genetics, Centre Mère Enfant Soleil, Laval University, Quebec City, QC, Canada; ^3^Department of Pediatric Neurology, Pediatric Neuromuscular Disorder, Centre Mère Enfant Soleil, Laval University, Quebec City, QC, Canada

**Keywords:** nemalin myopathy, neuromuscular disorder, alternative splicing, nebulin isoforms, nebulin, *NEB*

## Abstract

Nemaline myopathy is a rare disorder affecting the muscle sarcomere. Mutations in nebulin gene (*NEB*) are known to be responsible for about 50% of nemaline myopathy cases. Nebulin is a giant protein which is formed integrally with the sarcomeric thin filament. This complex gene is under extensive alternative splicing giving rise to multiple isoforms. In this study, we report a 6-year-old boy presenting with general muscular weaknesses. Identification of rod-shaped structures in the patient' biopsy raised doubt about the presence of a nemaline myopathy. Next-generation sequencing was used to identify a causative mutation for the patient syndrome. A homozygous deep intronic substitution was found in the intron 144 of the *NEB*. The variant was predicted by *in silico* tools to create a new donor splice site. Molecular analysis has shown that the mutation could alter splicing events of the nebulin gene leading to a significant decrease of isoforms level. This change in the expression level of nebulin could give rise to functional consequences in the sarcomere. These results are consistent with the phenotypes observed in the patient. Such a discovery of variants in this gene will allow a better understanding of the involvement of nebulin in neuromuscular diseases and help find new treatments for the nemaline myopathy.

## Introduction

Nemaline myopathies (NM) are a rare form of early-onset myopathy presenting at birth or early childhood with generalized muscle weaknesses and hypotonia. Incidence of NM in general population is estimated at one on 50,000 (Orphanet, https://www.orpha.net/consor/cgi-bin/index.php?lng=FR). Essentially the neck, facial, distal, and proximal muscles, as well as respiratory muscles are affected (1, 2). The spectrum of clinical phenotypes is wide, ranging from severe, intermediate, and typical congenital form to mild childhood or juvenile onset form. Presence of nemaline bodies observed in muscle histopathological biopsy of affected individuals are characteristics that are sometimes found in NM (3–5). These rod-like structures derive mainly from sarcomeric Z disc aggregates and thin filament proteins which are made visible by Gömöri trichrome staining (6). The presence of cap-like structures, disorganized myofibrils, thickened Z disks, as well as fiber-type disproportion are other histopathological characteristic features of NM (7–9). Autosomal dominant or recessive inheritance observed in *ACTA1* (10), *NEB* (11, 12), *TPM2* (13), *TPM3* (14, 15), *KBTBD13* (16), *CFL-2* (17), *KLHL40* (18), *KLHL41* (19), *LMOD3* (20), *MYPN* (21), *TNNT1* (22, 23), *TNNT3* (24), *MYO18B* (25), and *RYR3* (26) have been linked to different types of NM. Variations in the genes encoding the skeletal muscle α-actin (*ACTA1, OMIM* 102610) and nebulin (*NEB, OMIM* 161650) are the most common causes of this neuromuscular disorder. Usually, mutations in *ACTA1* gene are *de novo* dominant while known *NEB* variants are mostly recessive. Although a dominant mutation causing NM was recently identified in the *NEB* gene (27). Among the more than 3,000 of reported *NEB* variations in ClinVar and Leiden Muscular Dystrophy databases, only a small proportion (<6%) are pathogenic or likely pathogenic (https://www.ncbi.nlm.nih.gov/clinvar/?term=neb%5Bgene%5D, https://databases.lovd.nl/shared/variants/NEB/unique?search_var_status==”Marked”|=”Public”). Pathogenic variations in the *NEB* gene are mostly splice site, frameshift, and non-sense mutations. Approximately 50% of all cases of autosomal recessive NM are caused by variants in the *NEB* gene (28).

Nebulin was first discovered in 1980 (29). Its critical role in muscle function became evident when mutations in the gene were associated with autosomal recessive NM (30, 31). Nebulin gene encodes for one of the largest vertebrate proteins with a molecular weight of 600–900 kDa. The protein is mainly expressed in skeletal muscle but has also been detected in the brain and heart (32, 33). Nebulin is closely associated with the actin thin filament and anchors its C-terminal extremity in the Z disc of the muscle sarcomere. This major muscle protein has important role in the regulation of actin filament length, actin myosin interaction, and myofilament calcium sensitivity, and consequently in the regulation of muscle contraction (34, 35).

**Figure 1:**
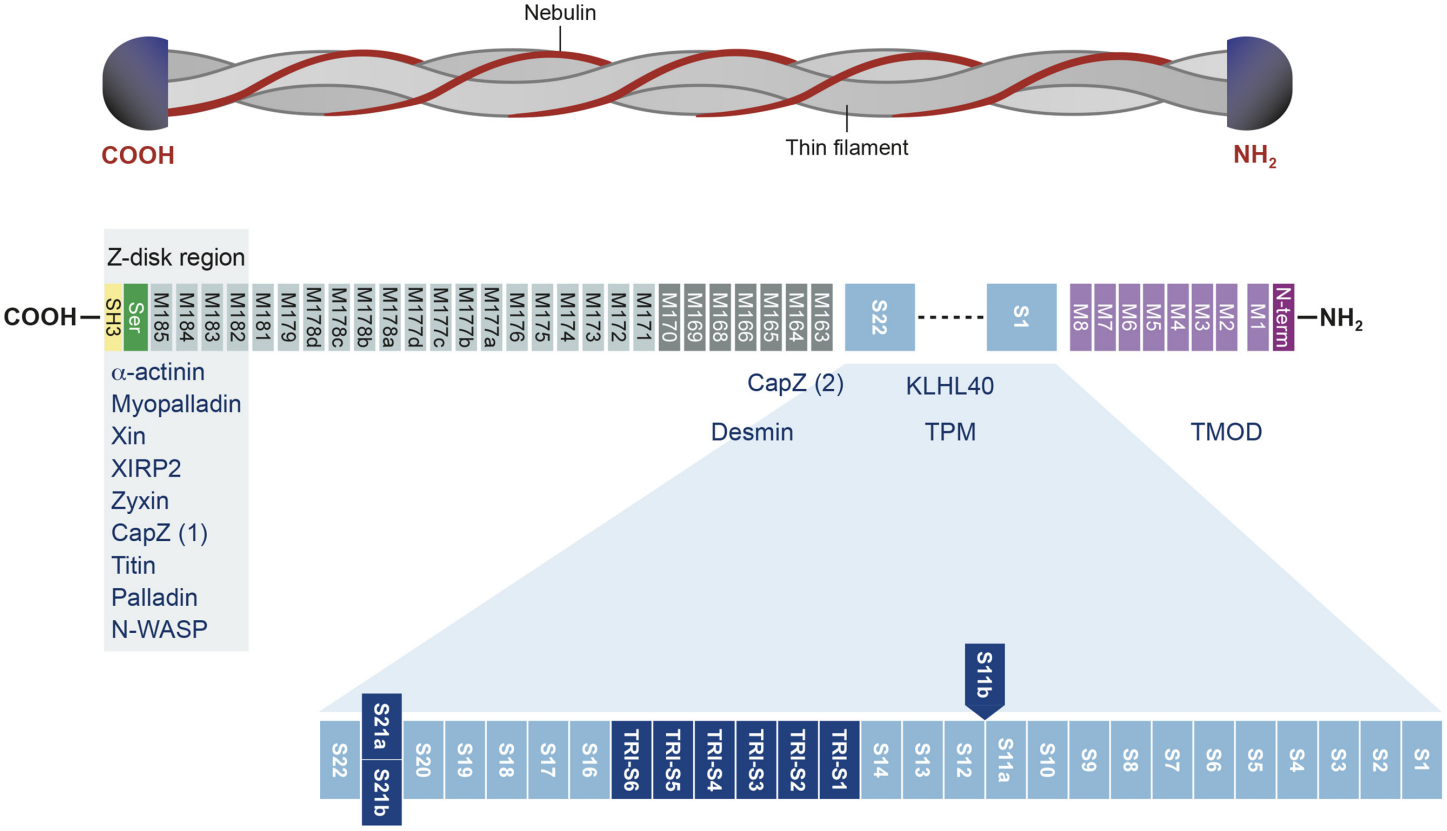
Structure of the nebulin protein and binding partners. Nebulin gene consists of multiple repeat modules (M) that contain actin-binding domain. The S21 super repeat encodes the mutually alternatively spliced exons 143 and 144 and contains the binding site for KLHL40. The C-terminal is anchored in the Z-disk region of the sarcomere and contains a conserved serine-rich homology domain (SH3). This terminal region binds many muscle proteins. Binding partners are written in blue. Adapted from Yuen and Ottenheijm (39), http://creativecommons.org/licenses/by/4.0/.

In humans, *NEB* is located on chromosome 2 (36) and consists of 183 exons, of which 42 are alternatively spliced, giving rise to the broad isoform diversity of nebulin. Exons 63–66, 82–105, 143–144, and 166–177 are alternative spliced exons, and exon 143 and 144 have not been detected in the same transcript (37, 38). The structure of the NEB protein is composed of simple motif repeats, of a central super repeat regions SR1 to SR22 made up of seven simple repeats each, and of a serine-rich SH3 domain in C-terminal (39). The repeat modules contain the essential conserved binding motifs for actin. Nebulin has multiple binding partners, for instance N-terminal region of NEB has been shown to bind tropomodulin. The central super repeat region is thought to interact with tropomyosin and KLHL40, and the SH3 domain located at the C-terminal interacts with a large number of proteins (Figure 1).

This large variety of transcripts makes nebulin one of the most complex genes involved in neuromuscular disorders because a mutation can selectively affect certain isoforms (30, 37). Since *NEB* gene is extensively spliced, frameshift mutations, for example, are likely to abolish expression of some nebulin isoforms, leaving others untouched, making functional effects difficult to predict. In fact, no human patient with *NEB* mutations causing total absence of the protein have been reported (40). To date, it seems that there is no mutation hotspot since mutations are distributed throughout the gene, although a deletion of the entire exon 55 was found to be a common founder mutation in an Ashkenazi Jewish population (41, 42).

Until now, there is no known effective treatment for nemaline myopathy. Tyrosine supplement has been proposed, but beneficial effects were not supported by subsequent studies in animal models and humans (43, 44). Supplementation with other amino acids was tested in zebrafish without clear positive effects (45). Relying on genetic analysis is sound to diagnose and better understand NM. Rapid advances in the field of next-generation sequencing allow the rapid discovery of new mutations responsible for this myopathy. In this study, we present a novel variant in the *NEB* gene of a young boy with nemaline myopathy.

## Materials and Methods

### Recruitment of Families and Ethic Statement

Families affected with a rare disease are recruited in an interdisciplinary research program designated “Programme de Recherche et Innovation Sur les Maladies rarES” (PRISMES) at the CHU de Québec-Laval University (CHU de Québec-UL) Research Center. PRISMES essentially aims to recruit pediatric patients and their affected/unaffected family members as a trio or more with the aim of investigating the genetic causes responsible for their diseases. Recruited patients are affected with rare neuromuscular, neurodegenerative, metabolic, or polymalformative syndrome, which remained undiagnosed at the molecular level. For this study, the affected boy was meeting our PRISMES project selection criteria along with his unaffected sister and his two unaffected parents. All samples from affected individuals and their families were obtained after approval by the “Comité d'éthique de la recherche (CER),” and all participants provided written informed consent prior to their enrolment. Research ethical board approval of the study design was obtained from the CER du CHUQ-UL.

### Biological Sample Collection

Ten milliliters of blood samples was drawn for all recruited individuals in the family. Half was used for genomic DNA extraction, and the other half was used for cellular immortalization. A quadricep skeletal muscle biopsy was surgically obtained during the clinical investigation at the CHU de Quebec. Histopathologic assessment was performed on sections of the muscle tissue. The remaining sample was frozen in liquid nitrogen and conserved at −80°C for subsequent analyses. Normal skeletal quadricep muscles pooled from four healthy individuals age 24, 30 78, and 87 were used as control.

### Library Preparation and Whole Exome Sequencing

DNA was extracted from 2 ml blood volume using QIAamp DNA Blood kit (Qiagen, Valencia, CA) according to the manufacturer's instructions. Libraries have been prepared from 3 μg of high-quality genomic DNA using SureSelect XT human All exon V6+UTR kit (Agilent Technologies, Santa Clara, CA). DNA was fragmented on a Covaris instrument (Covaris, Woburn, MA) and adaptor tagged to an average size of ~275–300 pb. Libraries were then subjected to exome capture. Three libraries with a unique index were pooled together in equimolar ratio and sequenced at a mean coverage of 100 × on an Illumina HiSeq2500 for paired-end 125 pb sequencing at both sites.

### Bioinformatics Analyses and Variant Filtering

Raw data were demultiplexed using Illumina's proprietary bcl2fastq to get to an open format. Then raw reads were trimmed using Trimmomatic (46) and mapped to human reference genome (hg19) using BWA (47). Duplicated reads were flagged using Picard MarkDuplicates and base score recalibration was performed using GATK BaseRecalibrator. Variant call was first performed on individual samples using Genome Analysis Toolkit (GATK) HaplotypeCaller before performing multisample joint aggregation and reannotation using GATK GenotypeGVCFs. Variants were functionally annotated based on data from SiFT (48), CADD (49), avsnp, Kaviar, ExAC, esp6500siv, 1000genomes, and PolyPhen 2 (50) using Annovar (51). Variant rarity was assessed with databases of variant frequencies in different populations from gnomAD, ExAC, and 1000Genomes. The availability of exome data from family individuals allowed identification of potential deleterious variants based on recessive, *de novo*, and compound heterozygote transmission modes. Additionally, a custom-automated bioinformatics pipeline built using GATK best practices and the Snakemake workflow, DNA-SEQ-GATK variant calling, was used to validate the previous bioinformatics analyses. For variant annotation, Ensembl Variant Effect Predictor (VEP) was used (52), and the results were visualized and filtered using the open-source SEQR platform.

### Cloning of Nebulin Fragments

To detect fragments spanning the mutation region, oligoprimer pairs specific for exons 142, 143, 144, and 145 of the nebulin gene were designed using GeneTool 2.0 software (Biotools Inc., CA) (Table 1). Nebulin fragments were amplified by PCR with cDNA synthesized from the patient quadricep muscle total RNA extract. Total RNA from normal skeletal muscle was used as control. The different PCR products were extracted on agarose gel, purified and cloned in blunt II-TOPO vector according to Invitrogen procedure (LifeTechnologies, Carlsbad, CA). Cloned plasmids were then transformed in TOP10-competent cells and amplified in LB-kanamycin culture media. Extracted DNA fragments were sequenced and analyzed using SnapGene viewer software.

**Table 1:** Primer sequences and gene description.

**Gene symbol**	**Description**	**GenBank**	**Size** **(pb)**	**Primer sequence 5**^**′**^ **→ 3**^**′**^ **S/AS**
NEB exons 146–148	Homo sapiens nebulin (NEB) exons 146–148	NM_001271208	236	AATACAACAAGGCCAAACCCAGAG**/**GTGGGCTTTGTTGGCTTCGTA
NEB exons 142–145	Homo sapiens nebulin (NEB) exons 142–145	NM_001271208	248–353	TGTTGCCGACTCTCCGATCA**/**CTTTTGTAGTACGCAGGTGTTCT
NEB exons 143–145	Homo sapiens nebulin (NEB) exons 143–145	NM_001271208	164–269	GCGGAAAGATAAATACCACCTG**/**CTTTTGTAGTACGCAGGTGTTCT
NEB exons 144–145	Homo sapiens nebulin (NEB) exons 144–145	NM_001271208	189	GAAAATATAAATCTAGTGCCAAG**/**CTTTTGTAGTACGCAGGTGTTCT

### Qantitative Real-Time PCR

Muscle biopsy was homogenized in Qiazol buffer (Qiagen, CA), and total RNA was extracted using the miRNeasy microkit (Qiagen, CA) following the manufacturer's instructions. First-strand cDNA synthesis was accomplished using 4 μg of RNA in a reaction containing Superscript IV, RnaseH-RT, oligo-dT_18_ (Invitrogen Life Technologies, Burlington, ON), random hexamers, dNTPs, and buffers. Oligoprimer pairs specific for exons 142 and 145 of the nebulin gene were designed using GeneTool 2.0 software (Biotools Inc., CA) (Table 1). Quadruplicate cDNA corresponding to 20 ng of total patient or control RNA was used to perform fluorescent-based real-time PCR quantification using the LightCycler 480 (Roche Diagnostics, Mannheim, DE). PCR reactions were as follows: 45 cycles, denaturation at 98°C for 10 s, annealing at 55°C for 10 s, elongation at 72°C for 20 s, and then 74°C for 5 s. A melting curve was performed to assess non-specific signal. Relative quantity was calculated using second derivative method and by applying the delta Ct method. Normalization was performed using the reference gene shown to be gene having stable expression levels from embryonic life through adulthood in various tissues: beta-2-microglobulin (B2M), hypoxanthine phosphoribosyltransferase 1 (HPRT1), and glyceraldehyde-3-phosphate dehydrogenase (GAPDH). Quantitative real-time PCR measurements were performed by the CHU de Québec Research Center (*CHUL*) Gene Expression Platform and were compliant with MIQE guidelines.

## Results

### Patient

**Figure 2:**
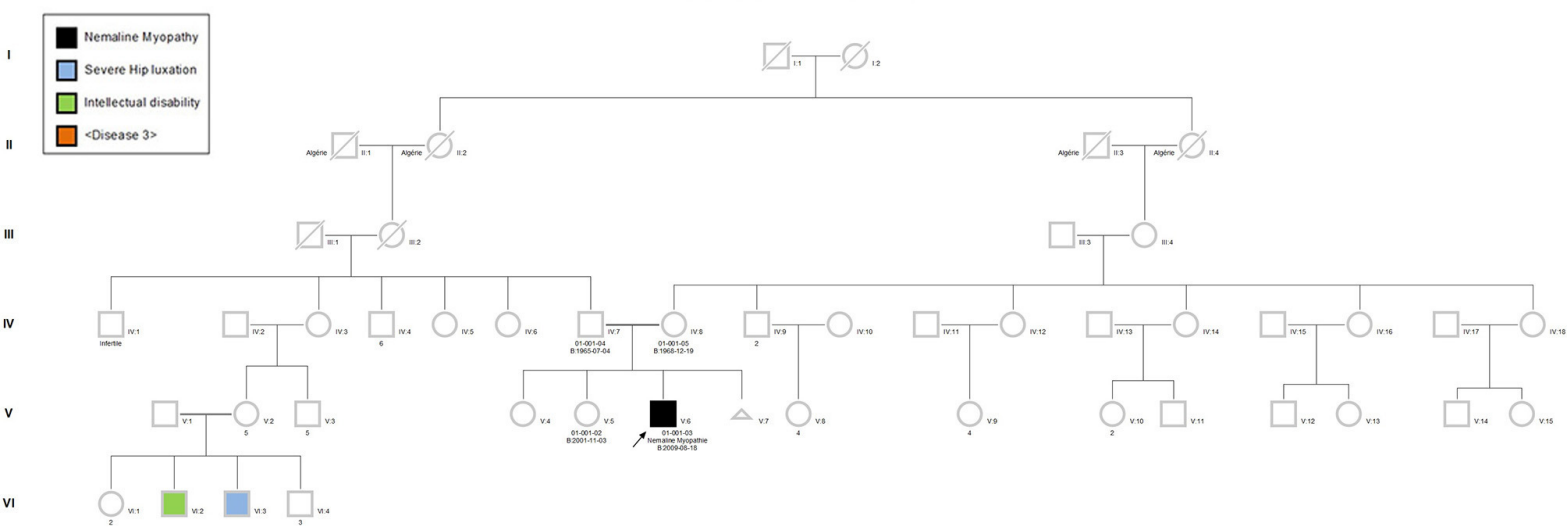
Pedigree of the patient's family. The proband (black square), his sister, father, and mother were recruited in the project. Due to the consanguinity, autosomal recessive type of inheritance was suspected. Pedigree symbols. Circle, female; square, male; black filled, affected; unfilled, non-affected.

**Figure 3:**
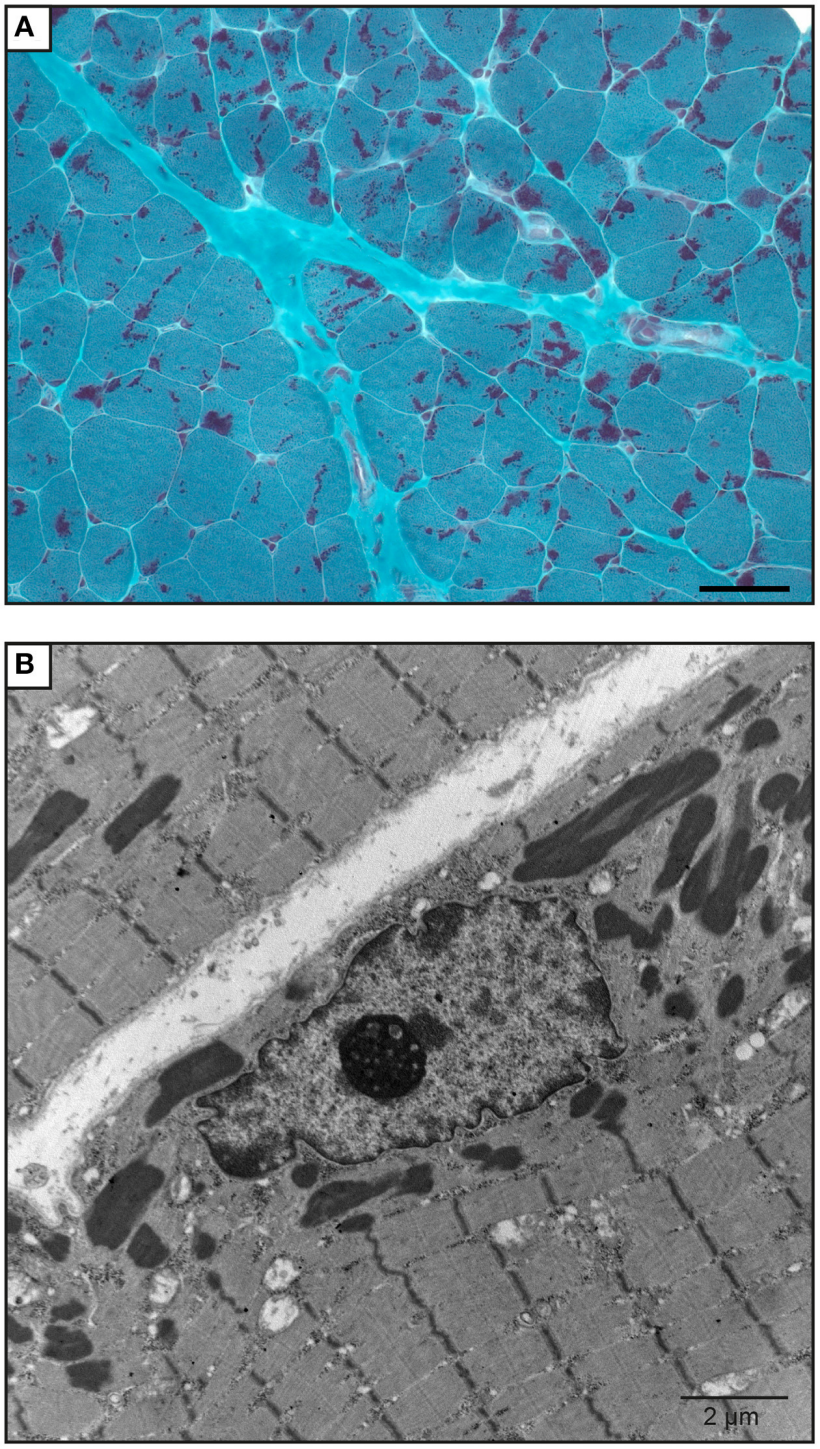
Muscle biopsy of the patient. **(A)** Gömöri trichrome staining of a quadricep muscle biopsy section shows mild anisomorphism fibers. More than 90% of the fibers have red stain inclusions typical of nemaline rods. Scale bar = 50 μm. **(B)** Electron microscopy image shows cytoplasmic rod-shaped nemaline bodies. No intranuclear rods were observed. Magnification, ×3,200; scale bar = 2 μm.

The proband was a 6-year-old boy who had muscle weaknesses. The first-year development showed a delay of gross motor functions; he walked at 15 months with support. At the age of 6, he was not able to run, jump, and walk on heels and had difficulties climbing the stairs. Clinical exam showed generalized muscle atrophy. He has thin prolonged myopathic face, mild bilateral ptosis, and high palate. Mild hyper-lordosis was present, and proximal and distal muscle weaknesses were noted. EMG/NCV registered proximal and distal myopathic features, and muscle creatine kinase level was within the reference range. He denied any swallowing problem neither orthopedic issues. Cardiac evaluation was normal. Parents were from Algeria and were closely related (Figure 2). Extensive genetic investigation including congenital myopathy gene panels, neuromuscular disease gene panels, and clinical exome were unsuccessful. Due to the negative results of the clinical genetic analysis, we proceeded with muscle biopsy, which showed presence of rod-shaped nemaline bodies that are indicative of nemaline myopathy (Figure 3). History of consanguinity prompted us to proceed with CGH-SNP array to identify homozygous blocks within the patient's genome. There were six large homozygous blocks, including well-known nemaline myopathy genes *NEB*: 1p13.3-q25.3; 2q11.2-q31.1; 5p13.2-q14.1; 11p14.2-p13; 16q22.1-q23.2; 21q11.2-q21.1.

### Genetic Results

**Figure 4:**
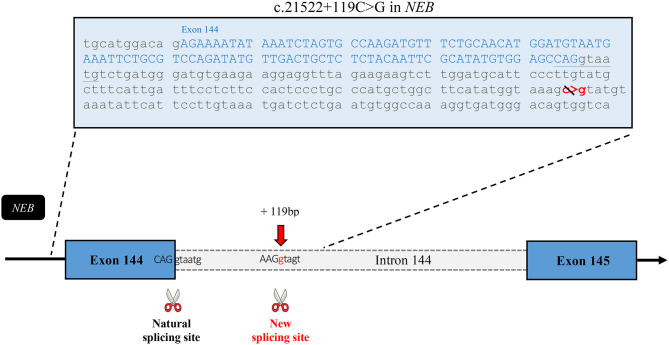
*In silico* analysis of the effects of the mutation. Sequence of the exon 144 followed by the first part of the intronic sequence that includes the mutation. This schematic representation of the affected region of the nebulin gene shows the new splice site predicted to be created by this intronic variant. According to Human Splicing Finder, the new site has a strength of 90.24^*^ in comparison with 85.75^*^ for the natural site. ^*^These numbers are relative consensus values established by the tool ranging from 1 to 100.

Following the inconclusive clinical investigation, the affected boy and his unaffected mother, father, and sister were recruited in PRISMES's project. Genome analysis was first initiated by a private company with impact to the regions within homozygous blocks. Meanwhile exome sequencing of the participants was performed at the NGS platform of the CHU de Quebec. Exome sequencing covered up to 125 bp of intronic part flanking the exons with excellent coverage. Variants were then analyzed through PRISMES's pipeline. Both PRISMES and clinical genetic analysis have identified a novel homozygous mutation NM_001271208.1: c.21522+119C>G in the intron 144 of *NEB* gene, which positively segregates in a family. This mutation is classified as a variant of uncertain significance according to recommendations of the American College of Medical Genetics and Genomics (ACMG). This deep intronic substitution, located at 119 bp following the end of exon 144 in the genome, is predicted by *in silico* tools to create a new splice site that could affect the natural splicing site of the same intron. Indeed, according to human splicing finder, the new donor splicing site created by the C > G substitution in the intron is as strong if not more than the natural splicing site at the end of exon 144 (Figure 4). These predictions led us to speculate that the splicing of the intron 144 could occur at both locations, leading to normal and altered isoforms. As mentioned, exons 143 and 144 are not found in the same isoforms. It is therefore very likely that perturbation in this critical region subject to alternative splicing could lead to change in isoform levels or functions.

### Analysis of Exons 142–145 Covering Region

**Figure 5:**
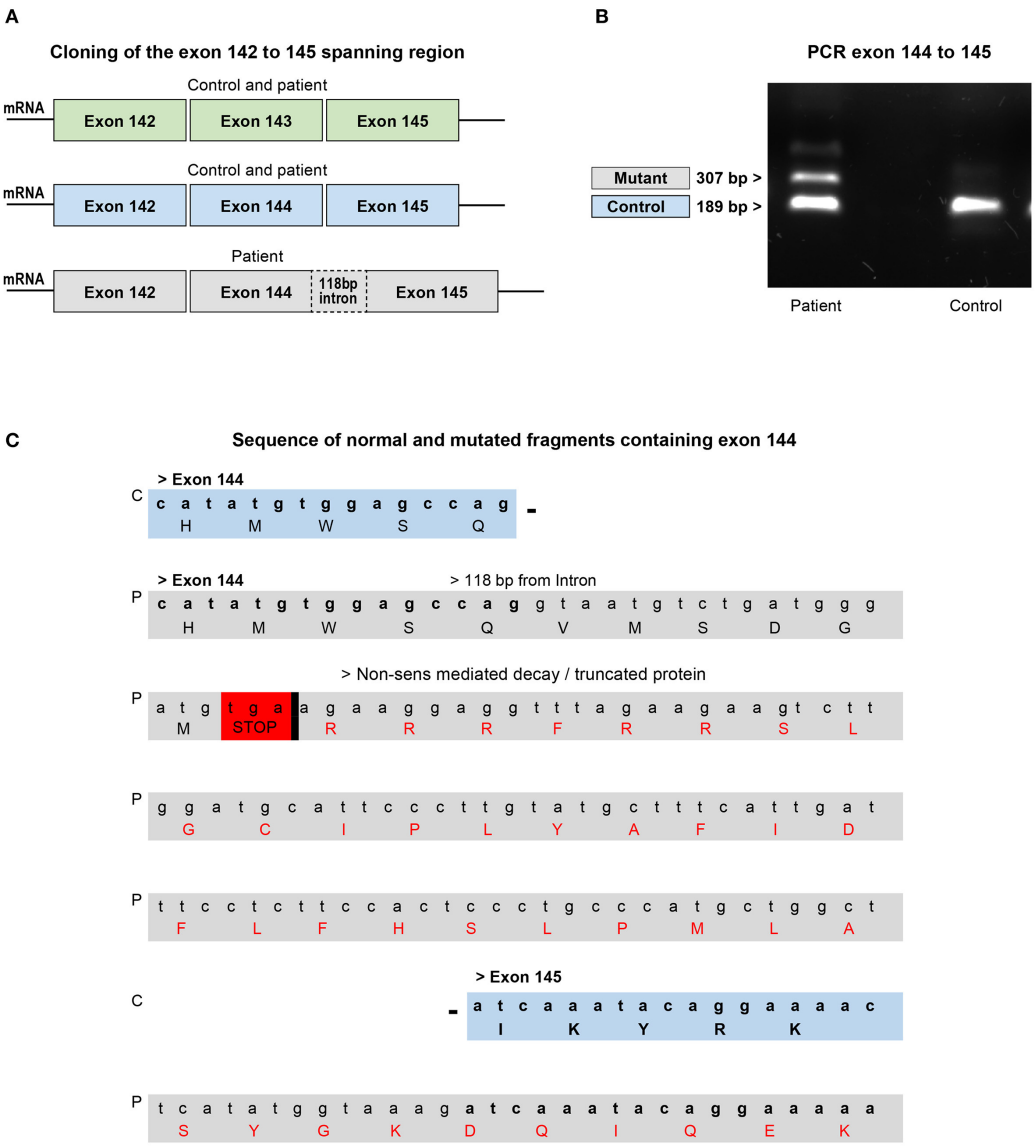
Analysis of cloned fragments ranging from exons 142–145 from the patient's biopsy or skeletal muscle control RNA. **(A)** Fragments with or without exons 143 and/or 144 found in the control and patient's biopsy. **(B)** Electrophoresis analysis of the PCR fragment of the exons 144–145 shows the presence of an additional band in the patient which corresponds to the intron containing fragment found in the biopsy. The smaller fragment found in both control and patient corresponds to the normal exons 144–145 region. **(C)** Comparison of part of the sequence of the normal and intron containing fragments with exon 144. The mutation disrupts the reading frame by introducing a premature termination codon that targets the mRNA for degradation or production of a truncated protein. C, control; P, patient.

To assess the effects of the mutation on this critical region, exons 142–145 of nebulin cDNA from patient biopsy and control were amplified by PCR and cloned. Analysis of sequencing data of 96 clones allowed, as predicted, detection of fragments with exon 143 or with exon 144 but never both at the same time. In the patient's biopsy, clone with intact exon 143 or 144 was also detected indicating that some transcripts are properly spliced. But interestingly, few fragments with exon 144 that include 118 additional nucleotides were found (Figure 5A). Indeed, electrophoresis analysis of amplified PCR fragments using primer selected in exons 144 and 145, showed an additional band of higher molecular weight in the patient's muscle cDNA (Figure 5B). These additional base pairs correspond to the nucleotide sequence upstream of the mutation in intron 144, meaning that in these isoforms, the new splicing site created by the mutation competed with the natural splicing site, leading to a pseudo exon. Analysis of this sequence using Open Reading Frame finder and ExPASy translate bioinformatics tools predicted that the addition of the 118-bp sequence disrupt the reading frame and introduce premature termination codon which would give a truncated protein of 7,180 amino acids instead of 8,560 (Figure 5C). This suggested that this mutant mRNA is more likely to become the target for degradation by non-sense-mediated decay (NMD) and that most of them are probably degraded before being translated into the protein (53, 54). In fact, very few clones with this partial intron retention was found among the hundred clones analyzed, indicating that this mutated isoform is probably very unstable. These transcriptional disturbances are most likely responsible to affect the level of nebulin expression. In order to assess relative quantities of the nebulin in the patient biopsy compared with those of the control pool, we performed quantitative real-time PCR on both skeletal muscle RNA samples.

### Comparative Analysis of RNA Expression in Patient and Control Muscle Biopsy

**Figure 6:**
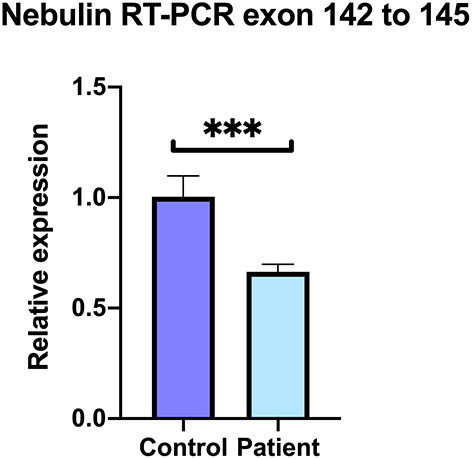
Decreased levels of nebulin expression in the patient's skeletal muscle. Quantitative real-time RT-PCR comparative analysis on the covering region from exons 142–145. Relative expression represented as fold ratio of nebulin fragments in the patient biopsy compared with control. Fold changes were normalized using the three reference genes beta-2-microglobulin (B2M), hypoxanthine phosphoribosyltransferase 1 (HPRT1), and glyceraldehyde-3-phosphate dehydrogenase (GAPDH). Data are represented as mean of the quadruplicate ± SD. ^***^P = 0.0005.

At least 20 different nebulin isoforms are reported among which 13 code for a protein (ENSEMBL, https://www.ensembl.org/Homo_sapiens/Gene/Summary?db=core;g=ENSG00000183091;r=2:151485336-151734487). To compare the levels of expression of nebulin transcripts in patient vs. control, quantitative RT-PCR analysis was performed on the patient's and control's skeletal muscle sample. Primer set were selected in the exons 142 and 145 of the nebulin to cover the affected region. Result shows that normalized relative expression of nebulin fragments 142–145 in the patient biopsy represented 69% of the control, meaning that there is a decrease of 34% of the expression of isoforms including fragments 142–145 (Figure 6). These results tend to suggest that some nebulin isoforms are affected by this mutation in intron 144.

As we have shown, the creation of a new splicing site in this mutated isoform disturbs the natural intron splicing and generates a pseudo exon susceptible to degradation or lead to a truncated protein. Impaired transcription of nebulin isoforms is therefore not so surprising. Indeed, the intron splicing and/or excision of the two mutually exclusive exons 143 and 144 are likely to be interdependent, and the mutation could lead to a defective alternative splicing in this critical region (55). The alternative splicing events in nebulin are numerous and interrelated. The production of the different isoforms follows a rigorous cascade of events. We can easily imagine that an obstacle to this chain of events will lead to perturbation in the isoforms transcription. Formation of a pseudo exon which makes pre-mRNA vulnerable for degradation, the presence of important regulatory sequence within the mutated intron, or the formation of an abnormal secondary structure in the pre-mRNA are all situations that may affect transcription (55, 56). Altogether, these data suggest that this novel homozygous mutation in *NEB* alters the expression levels of nebulin in this patient's skeletal muscle. It is therefore a possibility that this variant is related to the nemaline myopathy of this young patient.

## Discussion

PRISMES project has been launched in 2017. Since then, 118 families including probands and their affected or non-affected close relatives have been recruited in the project. Over the years, we had several cases presenting with neuromuscular disorders, but this young boy was our first case diagnosed with nemaline myopathy. Presence of rod-like accumulations in the biopsy in combination with the typical patient's phenotypes made the diagnostic of NM quite clear (28). The discovery of this intronic mutation in the *NEB* gene following the NGS sequencing of the patient and his family reinforces this idea.

During the last decade, more than 3,000 variants were found in different regions of the nebulin (Exome Variant server, https://evs.gs.washington.edu/EVS/). *NEB* is special for its giant size and for its high number of splicing events, giving rise to a multitude of different nebulin isoforms (57). Although various mutations were found all over the gene, it is logical to speculate that mutations affecting the splicing mechanisms would be harmful. In fact, in a study cohort of 159 families, Lehtokari et al. have shown that up to 34% of the mutations found in *NEB* gene were suspected to affect splicing sites (31). However, even if splicing site mutations are frequent, variants affecting the alternatively spliced exons of *NEB* are still rare (ClinVar, https://www.ncbi.nlm.nih.gov/clinvar/?term=neb%5Bgene%5D). We might think that this kind of mutation in *NEB* gene would be even more critical (58). To our knowledge, we report in this study the first homozygous mutation that is thought to disrupt the splicing of the alternatively spliced exon 144. To date, only two pathogenic or likely pathogenic mutations in exon 144 were reported: (ex144; c.21423del; p.Lys7141fs - ex144: c.21506C>A; p.Ser7169^*^) (31).

Our results suggested that the mutation in the intron 144 created a new splicing site strong enough to compete with the natural splicing site next to the mutation. The similar strength of the new and natural splicing sites can let us to believe that both sites could be used equally which could partly explain that many isoforms are not affected by the mutation. The relative diminution of nebulin isoforms in the biopsy compared with the normal control is likely to be attributed to the creation of an early stop codon generated by the frameshift, when the new splicing site is used. Most of the time, this situation is known to expose the mutant mRNA to degradation by non-sense-mediated decay. Several deep intronic mutations leading to inclusion of a pseudo exon have been reported in patients affected by multiple disorders (53, 54).

In our study, we have also found few isoforms with a larger pseudo exon 144, including a small sequence belonging to the intron that were apparently not degraded. However, those isoforms are predicted to be truncated and lack the 1,380 last amino acid of the nebulin protein. These shortened transcripts are very likely to be non-functional since they lacked the C terminal portion of the gene which encodes the SH3 domain (59). The binding of multiple essential partners of nebulin have been found to be regulated by this highly conserve serine-rich SH3 domain (39). Among those we found are α-actinin, myopalladin, CapZ, titin, and many other major players of the muscle sarcomere (Figure 1). C-terminus of nebulin anchors the Z-disk and also contributes to stabilization and length regulation of the thin filament (40). It has been shown that thin filament dysregulation resulting of a mutation in *NEB*, can contribute to muscle weakness in patients with nemaline myopathy (60, 61).

As the splicing of the two mutually exclusive exons 143 and 144 are likely to be related, it makes sense that the mutation in intron 144 affects these selective splicing events (55). The regulation and role of these exons in developmental and adult muscle fibers were reported to be important (62). Indeed, these exons are alternatively spliced depending on the muscle type and the developmental stage. Both isoforms have different charges and hydrophobicities suggesting they may have different functions. Lam et al. reported that nebulin containing exon 144 is present early in myogenesis while the one containing isoform 143 appears at later stages of the muscle development. Moreover, in human skeletal quadriceps, protein with exon 143 is expressed in fast fibers while almost absent in slow fibers (37, 63, 64). These exons also encode one domain of the S21 super repeat region of *NEB* gene that was shown to bind the KLHL40 protein (Figure 1). Mutation in *KLHL40* was associated with NM and is thought to be involved in stabilization of the thin filament and regulation of nebulin level (18, 65). These different functions led us to assume that in addition to a decrease in the global expression levels, a perturbation in the ratio of these isoforms would have negative consequences. A study of the isoforms of the giant muscle titin gene showed that not only the level of the different isoforms are important, but their ratio also appears to be crucial, among other things, during heart development (66).

The alternative splicing mechanistic of the nebulin is very complex and not well-understood. In their review, Rita Vaz-Drago and colleagues discussed how multiple types of splicing dysregulation may be caused by deep intronic mutation. More frequently, the degradation of the pre-mRNA and also the binding modification of splicing regulatory protein in the intron or formation of abnormal pre-mRNA secondary structure can disrupt the splicing event (53). Indeed, many studies show that a single regulatory element mechanism can regulate several splicing events on proximal and distal regions of a RNA molecule (55, 56).

This study suggests that although a good percentage of isoforms are still expressed in the patient, a decrease or disturbance in their ratios could have an impact on the proper function of this protein. Future discovery of other variants in the *NEB* gene and especially the one that are in alternatively spliced region will allow a better understanding of this complex giant protein. Indeed, finding of variants affecting the alternative splicing of nebulin will make it possible to know more about the function of the different isoforms and allow us to clarify their respective implications in congenital nemaline myopathy.

## Data Availability Statement

The datasets presented in this study can be found in online repositories. The names of the repository/repositories and accession number(s) can be found below: NCBI BioProject PRJNA702801.

## Ethics Statement

The studies involving human participants were reviewed and approved by Comité d'éthique de la recherche (CER) du CHU de Quebec, Université Laval. Written informed consent to participate in this study was provided by the participants' legal guardian/next of kin.

All samples from affected individuals and their families were obtained after approval by the “Comité d'éthique de la recherche (CER) du CHU de Quebec-Université Laval” and all participants provided written informed consent prior to their enrolment.

The protocol “Programme de Recherche et Innovation Sur les Maladies rarES” (PRISMES) approved by the CER at the “CHU de Québec-Université Laval (CHUQC-UL)” include the consent for data publication.

## Author Contributions

NL: study and manuscript design, performed the experiments, results analysis, and wrote the paper. BL: medical follow-up and diagnosis of the patient, variants and genetic analysis, and thorough reviewed the manuscript. ST: bioinformatic analysis (upload and analysis of sequencing data) and reviewed the manuscript. NR: patient and family recruitment, and reviewed the manuscript. YL: biobank management, DNA extraction, and variants analysis. AD: director of the bioinformatics platform, and supervised bioinformatic analysis. NC: medical follow-up of the patient, biopsy images follow-up, and reviewed the manuscript. SR: study director, suppervized the project, and reviewed the manuscript. All authors contributed to the article and approved the submitted version.

## Conflict of Interest

The authors declare that the research was conducted in the absence of any commercial or financial relationships that could be construed as a potential conflict of interest.
